# Content overlap of 91 dystonia symptoms among the seven most commonly used cervical dystonia scales

**DOI:** 10.1007/s10072-023-07157-1

**Published:** 2023-11-01

**Authors:** Adrian Andrzej Chrobak, Jakub Rusinek, Małgorzata Dec-Ćwiek, Karolina Porębska, Marcin Siwek

**Affiliations:** 1 Department of Adult Psychiatry, Chair of Psychiatry, Jagiellonian University Medical College, Kraków, Poland; 2 Students’ Scientific Group of Neurology at the Department of Neurology, Jagiellonian University Medical College, Kraków, Poland; 3 Department of Neurology, Jagiellonian University Medical College, Kraków, Poland; 4 Department of Affective Disorders, Chair of Psychiatry, Jagiellonian University Medical College, Kopernika 21a St., 31-501 Kraków, Poland

**Keywords:** Cervical dystonia, Clinical scales, Nonmotor symptoms, Movement disorders

## Abstract

**Introduction:**

Dystonia is a movement disorder characterized by sustained or intermittent muscle contractions. Cervical dystonia (CD) is the most common focal dystonia. There are several instruments assessing the symptoms of CD. However, different scales assess different features which may lead to poor patient evaluation.

**Aim:**

The aim of the study was to evaluate the degree of overlap of most often used CD rating scales identified by the literature review.

**Methods:**

A thorough search of the Medline database was conducted in September 2021. Then the frequency of each scale was calculated, and 7 most common scales were included in the content overlap analysis using Jaccard index (0 – no overlap, 1 – full overlap).

**Results:**

Toronto Western Spasmodic Torticollis Rating Scale (TWSTRS), Tsui score, Burke-Fahn-Marsden Dystonia Rating Scale (BFMDRS), Cervical Dystonia Impact Profile 58 (CDIP-58), Craniocervical Dystonia Questionnaire 24 (CDQ-24), Cervical Dystonia Severity Rating Scale (CDSS), Cervical Dystonia Severity Rating Scale (DDS) and The Dystonia Non-Motor Symptoms Questionnaire (DNMSQuest) were the most common scales. 91 CD symptoms were distinguished from 134 items used in the scales. The mean overlap among all scales was 0.17. 52 (62%) symptoms were examined by more than one scale. The CIDP-58 captured the highest number of symptoms (63.0%), while the CDSS captured the lowest number (8.0%). None of the symptoms were examined by seven instruments.

**Conclusions:**

There was a very weak overlap among scales. High inconsistency between the scales may lead to highly different dystonia severity assessment in clinical practice. Thus, the instruments should be combined.

## Introduction

Dystonia is the third most common movement disorder [1, 2]. It is characterized by sustained or intermittent muscle contractions causing abnormal movements or postures [3]. Currently, dystonia is classified along two axes: clinical characteristics, including age at onset, body distribution, temporal pattern and associated features (additional movement disorders or neurological features); and etiology, which includes nervous system pathology and inheritance [3]. Focal dystonia, which affects only one body region is the most common. Cervical dystonia (CD) is the one with the highest prevalence (from 57/1mln to 4000/1mln) [4]. Over the years multiple scales assessing symptoms and severity of CD were developed. The most popular ones are Toronto Western Spasmodic Torticollis Rating Scale (TWSTRS) [5]; Tsui scale [6]; Burke-Fahn-Marsden Dystonia Rating Scale (BFMDRS) [7]; Cervical Dystonia Impact Profile 58 (CDIP-58) [8]; Craniocervical Dystonia Questionnaire 24 (CDQ-24) [9]; Cervical Dystonia Severity Rating Scale (CDSS) [10]; Dystonia Discomfort Scale (DDS) [11]; The Dystonia Non-Motor Symptoms Questionnaire (DNMSQuest) [12]; Unified Dystonia Rating Scale (UDRS) [13] etc.

However, different scales focus on different features which might result in a poor assessment of disease severity. This problem mainly applies to non-motor symptoms (NMS) of dystonia. For instance, not all of the scales evaluate NMS as some of them were developed before the subject emerged [14]. In recent decades NMS have been the subject of extensive research – the most popular symptoms are: sleep disorders, anxiety and tiredness [15, 16]. Until now, there is no universal tool to assess them. There is a possibility of overlooking the symptoms by using one specific questionnaire.

Eiko Fried (2017) proposed a methodology for evaluating different assessment tools in order to designate the level of overlap of symptoms across the questionnaires [17]. Such analysis might give valuable information about the reproducibility and generalization of findings made by one tool. When the overlap is poor, it might indicate that the symptoms assessed by one tool are idiosyncratic and cannot be found by another tool – putting in doubt the comparability of different studies. Moreover, this methodology has been already used in several different studies [18–21]

The aim of our study was to evaluate the degree of overlap of most often used CD rating scales which were identified by the literature review. Furthermore, we gave recommendations on which scales should be combined to give more specific information about dystonia severity in research.

## Materials and methods

### Literature review

A thorough search of the Medline database was conducted by one researcher in September 2022. No language or date filters were applied. The query was as follows:

*"Torticollis"[MeSH Terms] AND ("scale s"[All Fields] OR "scaled"[All Fields] OR "scaling"[All Fields] OR "scalings"[All Fields] OR "weights and measures"[MeSH Terms] OR ("weights"[All Fields] AND "measures"[All Fields]) OR "weights and measures"[All Fields] OR "scale"[All Fields] OR "scales"[All Fields] OR ("questionnair"[All Fields] OR "questionnaire s"[All Fields] OR "surveys and questionnaires"[MeSH Terms] OR ("surveys"[All Fields] AND "questionnaires"[All Fields]) OR "surveys and questionnaires"[All Fields] OR "questionnaire"[All Fields] OR "questionnaires"[All Fields])).*

The inclusion criteria:

- assessing the symptoms of CD with the usage of a specific scale;
- patients ≥ 18 years old.

The exclusion criteria:

- type of article: meta-analyses, systematic review, literature review, case study;
- usage of scales which are not specific for dystonia assessment;
- non-cervical types of dystonia;
- studies developing a scale.

**Fig. 1:**
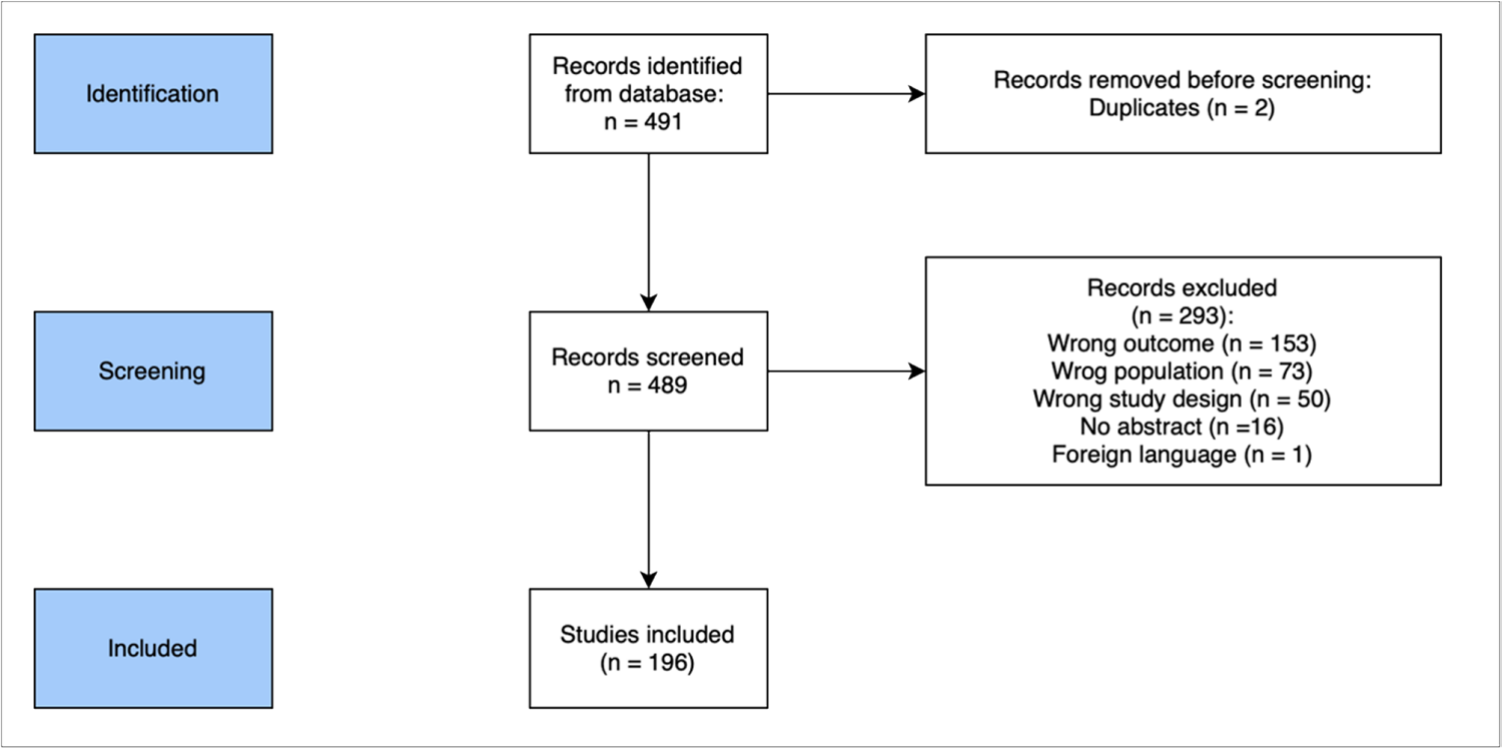
Flowchart of the inclusion process

Overall, 196 studies that fulfilled above mentioned criteria were included. Figure 1 presents a flow diagram representing the procedure of finding, excluding and selecting publications for further analyses. The frequency of each scale in the articles was calculated and the seven most common ones were included in the analysis. The decision of choosing the seven most popular used scales was based on the methodology of our previous study [18].

### CD scales

The chosen scales were: 20-item TWSTRS [5]; 7-item Tsui scale [6]; 8-item BFMDRS (8 items because only the part considering neck and disability was analyzed) [7]; 58-item CDIP-58 [8]; 24-item CDQ-24 [9]; 3-item CDSS [10]; 14-item DNMSQuest [12]. Table 1 presents the internal consistency, interrater and test-retest reliability of the tools. Although the frequency of DDS [11] was higher than DNMSQuest (1.5% vs. 1.0%) we decided to include DNMSQuest instead of DDS. Firstly, DDS is a scale that assesses only the patient’s experience related to the disease rated from 0 (no complaints) to 100 (maximum subjective severity of the untreated condition). That is why it is much different from other evaluated scales and thus cannot be compared with them. Secondly, DNMSQuest is a relatively new scale assessing NMS and it is not as well studied as other scales.

**Table 1:** Internal consistency interrater and test-retest reliability of analyzed tools. Toronto Western Spasmodic Torticollis Rating Scale (TWSTRS); Burke-Fahn-Marsden Dystonia Rating Scale (BFMDRS); Cervical Dystonia Impact Profile 58 (CDIP-58); Craniocervical Dystonia Questionnaire 24 (CDQ-24); Cervical Dystonia Severity Rating Scale (CDSS); The Dystonia Non-Motor Symptoms Questionnaire (DNMSQuest)

Scale	Internal consitency	Interrater reliability	Test-retest reliability
TWSTRS [5]	-	0.76—0.98^b^ [22]	-
Tsui scale [6]	-	0.86^c^	-
BFMDRS [7]	-	0.85—0.96^c^	0.98/0.99^c^
CDIP-58 [8]	≥ 0.92^a^ [23]	-	≥ 0.83^e^ [23]
CDQ-24 [9]	0.77—0.89^a^	-	≥ 0.90^e^
CDSS [10]	-	0.79^d^ for first evaluation 0.86^d^ for second evaluation	0.94^d^
DNMSQuest [12]	-	-	0.995^e^

^a^Cronbach’s alpha

^b^Kendall's coefficient

^c^Correlation coefficient

^d^Kappa value

^e^Test-retest intraclass correlations

### Content analysis

Based on the Fried (2017) methodology we adjusted the number of items in each scale [17]. The items were combined if they analyze the same symptom, in order to avoid biasing further analyses. The 8-item BFMDRS was reduced to 6 items. “Dressing”, “hygiene” and “feeding” were combined as one item assessing “limited daily activities”.

58-item CDIP-58 was reduced to 48 items. “Tension in the neck” and “tightness in the neck” were combined as one item assessing unpleasant feeling of tension in the neck. “Aching in shoulders” and “pain in shoulders” were combined as one item assessing pain in shoulders. Similarly to BFMDRS, items such as: “carrying light objects”, “chores”, “cooking”, “getting tired doing light activities”, “cleaning the house”, “limits in type of work”, “heavy chores”, “carrying heavy objects”, “getting tired doing demanding activities” were combined as “limited daily activities”. In our opinion, the analysis of specific activities mentioned in each scale would bias the analysis while those items evaluate a similar issue.

24-item CDQ-24 was adjusted to 23 items. “Feeling down or depressed” and “feeling sad” were combined as “feeling down”.

On the other hand, in TWSTRS we added one item “head movements” to make a comparison more reliable. Original TWSTRS evaluates head movements in detail but it lacks the general category describing them. Therefore, adjusted TWSTRS had 21 items.

After adjusting the scales, the maximum number of 91 symptoms were identified, and they were used in content analysis.

The categorization of items was also based on Fried’s (2017) methodology [17]. Items were treated as disparate if they distinctly differed from each other and they were considered equivalent if they evaluate the same symptom. For instance, “do you have any speech problem?” from DNMSQuest and “speech” from BFMDRS were considered as equivalent as both of them evaluated the same symptom – speech problems. On the other hand, “straining in the neck” and “stiffness in the neck” were not considered equivalent as those are different sensations. The symptom was defined as idiosyncratic if it appears in only one instrument.

### Statistical analysis

Firstly, each symptom was evaluated, and the decision was made if it appears specifically in the scale or the symptom is featured generally (indirectly) or is not featured at all. Secondly, the overlap of CD symptoms between the scales was calculated with the use of the Jaccard Index. It is a similarity coefficient for binary data (to run this analysis symptoms were categorized separately from previous analysis as present (1) or not (0) in the scale). The coefficient ranges from 0 (no overlap) to 1 (complete overlap) [17]. It was calculated by $\frac{s}{{u}_{1}+{u}_{2}+s}$, where *s* is the number of items two scales share and *u*_*1*_ and *u*_*2*_ stand for the number of items that are unique to each of the scales. Criteria of Jaccard Index power were as follows: very weak 0.00 – 0.19, weak 0.20 – 0.39, moderate 0.40 – 0.59, strong 0.60 – 0.79, very strong 0.80 – 1.00 [17, 24]. Moreover, the symptoms were divided into seven categories: motor symptoms, sensory symptoms, disability, sleep disturbances, gait disturbances, emotions, and social interactions. Then, the rate of symptoms in each category in each scale was calculated. Analyses were conducted with the code supplied in [17] with the usage of R software [25].

## Results

**Fig. 2:**
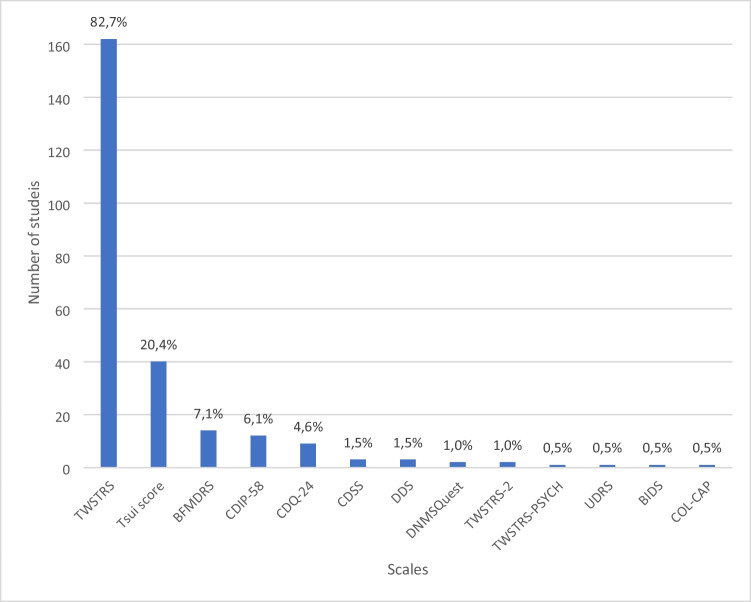
The number of scales in the studies. Toronto Western Spasmodic Torticollis Rating Scale (TWSTRS) [5]; Tsui scale [6]; Burke-Fahn-Marsden Dystonia Rating Scale (BFMDRS) [7]; Cervical Dystonia Impact Profile 58 (CDIP-58) [8]; Craniocervical Dystonia Questionnaire 24 (CDQ-24) [9]; Cervical Dystonia Severity Rating Scale (CDSS) [10]; Dystonia Discomfort Scale (DDS) [11]; The Dystonia Non-Motor Symptoms Questionnaire (DNMSQuest) [12]; Toronto Western Spasmodic Torticollis Rating Scale 2 (TWSTRS-2); Unified Dystonia Rating Scale (UDRS) [13]; Beth Israel Dystonia Screen (BIDS) [26]; Collum-caput concept (COL-CAP) [27]

From the included articles most commonly used scales were TWSTRS (in 162 articles), Tsui scale (in 40 articles), BFMDRS (in 14 articles), CDIP-58 (in 12 articles), CDQ-24 (in 9 articles), CDSS (in 3 articles), DDS (in 3 articles) and DNMSQuest (in 2 articles). Detailed information about the number of articles in which each scale was used can be found in Fig. 2.

**Fig. 3:**
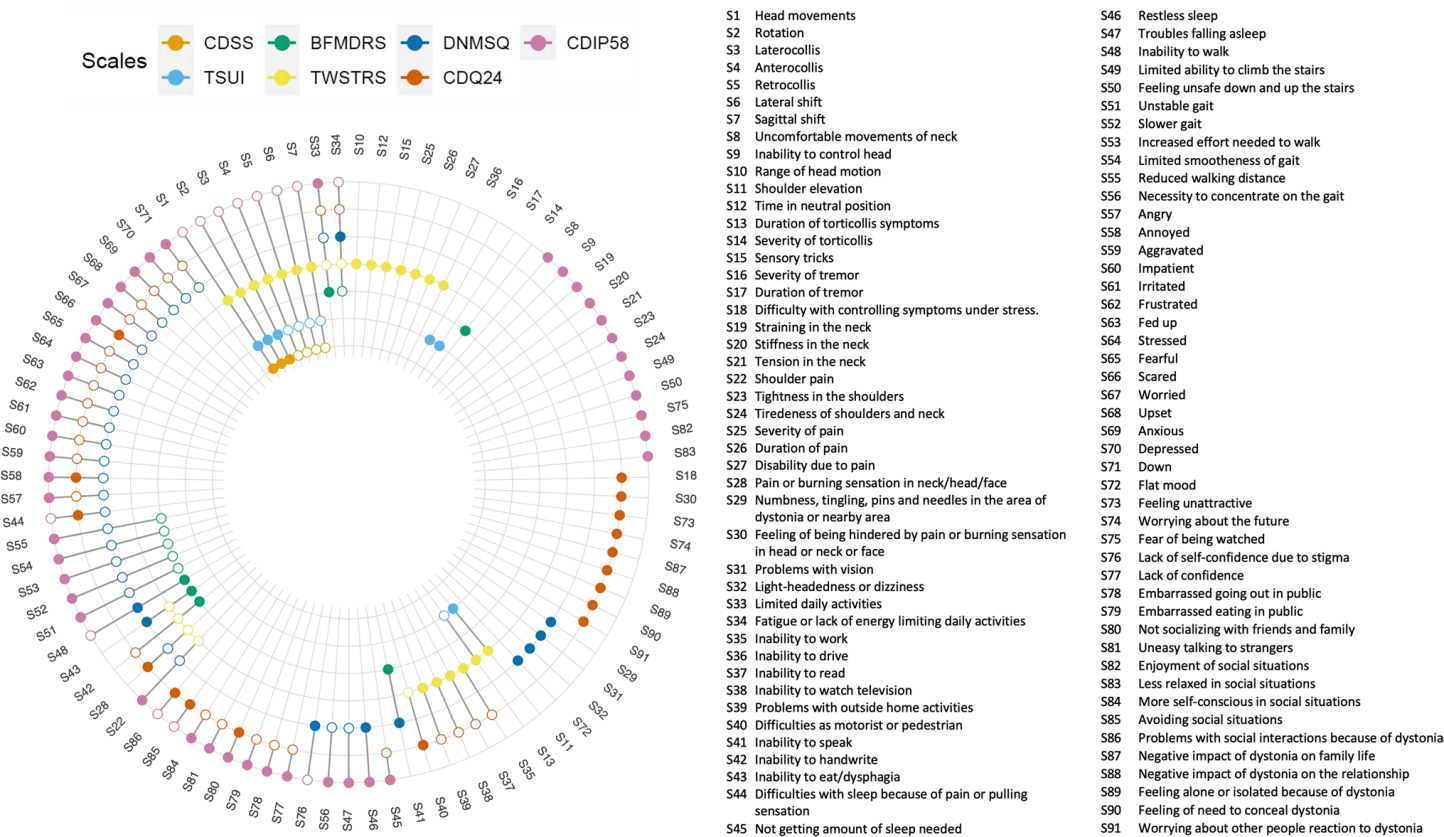
Presence of 91 dystonia symptoms in 7 most commonly used scales. Colored circles indicate that a symptom is directly assessed by the scale, while empty circles indicate that a scale only measures a symptom indirectly

The analysis extracted 91 distinct CD symptoms (Fig. 3). 52 (62%) symptoms are examined by more than one scale. Whereas none of the symptoms appear in six or seven instruments simultaneously (Table 2). Furthermore, each symptom is present in two instruments on average.

**Table 2:** The number of symptoms found in most commonly used scales. For example, 35 items, presenting 38% of all analysed cervical dystonia-related symptoms, are present in one instrument, while none of the items is present in every analysed scale

Number of items	Percent of all symptoms	Number of instruments
35	38%	1
21	23%	2
26	29%	3
7	8%	4
2	2%	5
0	0%	6
0	0%	7

In Table 3 the analysis of specific, compound and idiosyncratic symptoms can be found. CDIP-58 consists of the highest number of idiosyncratic symptoms (12 – which is 21% of items in this scale). CDSS is the only instrument that has no idiosyncratic symptoms. CDSS also analyses the lowest percentage of symptoms – 8% of 91 distinct symptoms. On the other hand, CDIP-58 examines the highest percentage of symptoms – 63%.

**Table 3:** The percentage of the specific, compound and idiosyncratic symptoms gathered in seven analysed tools, as well as the percentage of all 91 disparate items they capture

Scale	Specific symptoms	Compound symptoms	Number of idiosyncratic items:	Percent of idiosyncratic items	Sum of items:	Original number of scale items:	Adjusted number of symptoms per scale:	Scale captures x% of all 91 disparate items:
TWSTRS	74%	26%	7	26%	27	20	21	30%
Tsui	55%	45%	2	18%	11	7	7	12%
BFMDRS	50%	50%	1	8%	12	8	6	13%
CDIP-58	77%	23%	12	21%	57	58	48	63%
CDQ-24	40%	60%	9	21%	43	24	23	47%
CDSS	43%	57%	0	0%	7	3	3	8%
DNMSQ	28%	72%	4	11%	36	14	14	40%

TWSTRS is the instrument that analyses the highest number of symptoms in the motor symptoms category – 12 (67%) and in the disability category – 10 (91%). CDIP-58 captures the highest number of sensory symptoms, sleep disturbances and gait disturbances (six (43%), four (100%) and nine (100%), respectively). CDQ-24 consists of the highest number of symptoms related to emotions and social interactions (17 (94%) and 13 (76%), respectively). Detailed information about the symptom categories can be found in Table 4.

**Table 4:** The number of symptoms in each category examined by each scale. Toronto Western Spasmodic Torticollis Rating Scale (TWSTRS); Burke-Fahn-Marsden Dystonia Rating Scale (BFMDRS); Cervical Dystonia Impact Profile 58 (CDIP-58); Craniocervical Dystonia Questionnaire 24 (CDQ-24); Cervical Dystonia Severity Rating Scale (CDSS); The Dystonia Non-Motor Symptoms Questionnaire (DNMSQuest)

Category of symptoms	Number of symptoms overall	TWSTRS	Tsui scale	BFMDRS	CDIP-58	CDQ-24	CDSS	DNMSQuest
Motor symptoms	18	12 (67%)	11 (61%)	1 (6%)	9 (50%)	1 (6%)	7 (39%)	0 (0%)
Sensory symptoms	14	5 (36%)	0 (0%)	0 (0%)	6 (43%)	2 (14%)	0 (0%)	5 (36%)
Disability	11	10 (91%)	0 (0%)	5 (45%)	2 (18%)	8 (73%)	0 (0%)	4 (36%)
Sleep disturbances	4	0 (0%)	0 (0%)	0 (0%)	4 (100%)	2 (50%)	0 (0%)	3 (75%)
Gait disturbances	9	0 (0%)	0 (0%)	6 (67%)	9 (100%)	0 (0%)	0 (0%)	7 (78%)
Emotions	18	0 (0%)	0 (0%)	0 (0%)	15 (83%)	17 (94%)	0 (0%)	16 (89%)
Social interactions	17	0 (0%)	0 (0%)	0 (0%)	12 (71%)	13 (76%)	0 (0%)	1 (6%)

The mean overlap among all scales is 0.17 which is interpreted as a very weak similarity between the instruments [24]. Mean overlaps of single scales range from 0.09 (BFMDRS) to 0.22 (CDIP-58). The mean overlap is very weak for all scales apart from CDIP-58 (weak mean overlap). The overlap among specific scales ranges from 0.00 to 0.64 (Table 5).

**Table 5:** Overlap of the item content of 7 dystonia scales. The Jaccard Index ranges from 0 (no overlap) to 1 (total overlap)

	TWSTRS	TSUI	BFMDRS	CDIP58	CDQ24	CDSS	DNMSQ
TWSTRS	1,00	0,31	0,11	0,14	0,15	0,26	0,09
TSUI	0,31	1,00	0,00	0,11	0,00	0,64	0,00
BFMDRS	0,11	0,00	1,00	0,13	0,06	0,00	0,26
CDIP58	0,14	0,11	0,13	1,00	0,37	0,12	0,45
CDQ24	0,15	0,00	0,06	0,37	1,00	0,00	0,32
CDSS	0,26	0,64	0,00	0,12	0,00	1,00	0,00
DNMSQ	0,09	0,00	0,26	0,45	0,32	0,00	1,00
Mean overlap	0,18	0,18	0,09	0,22	0,15	0,17	0,19

The correlation between the mean Jaccard coefficient of each scale (the mean overlap a scale with all others) and the length of the scale is 0.61 for the number of specific symptoms captured and 0.54 for the adjusted scale length.

## Discussion

In our study, we have conducted a literature search and gathered 196 studies from which we have chosen the seven most commonly used instruments examining CD symptoms. According to the literature, TWSTR is the most popular rating tool used in CD research (82.7%). The second one is Tsui scale (20.4%). 91 disparate symptoms were identified. The mean overlap among all tools was very weak (0.17). As presented in Table 2 38% of symptoms were present in only one instrument while none of the symptoms appeared in all of them. What is more, we conducted the analysis of the categories of symptoms which revealed that some scales do not mention specific categories. It can be found that the most popular scale (TWSTRS) mainly focuses on motor symptoms and disability while there are no items evaluating psychological status. Tsui scale and CDSS also analyse only motor disturbances. On the other hand, BFMDRS focuses mainly on gait and disability. There is also a group of scales (CDIP-58, CDQ-24 and DNMSQuest) focusing on NMS, omitting the motor symptoms. Furthermore, we showed there is a moderate correlation between the Jaccard coefficient and the length of the scale showing that scales with a bigger amount of items have higher level of similarity to other questionnaires.

To our best knowledge, this is the first study to analyse the content overlap of the scales used in CD research. Previously published comparisons of scales focused on the clinical applicability, validity and descriptive analysis of the symptoms measured by the scales [28–30]. In some studies, CD symptoms were examined by more than one scale. For instance, Tarsy used TWSTRS and Tsui scale to assess the dystonia severity for the same patients in the study about botulinum toxin efficacy and found a significant positive correlation between the reduction of two scales scores after the treatment [31]. Considering our analysis, those scales analyse mainly motor symptoms which might explain a similar result, regardless of the weak overlap of the questionnaires (0.31). On the other hand, Tomic et al. presented a significant correlation between disability measured by TWSTRS and subscales of CDQ-24 [32]. However, considering the very weak overlap between TWSTRS and CDQ-24 (0.15) the symptoms assessed by CDQ-24 might be overlooked while using only TWSTRS. As was shown by Jost et al. TWSTRS is the main scale used in randomized controlled trials regarding botulinum toxin efficacy which is in line with our literature review in which it was the most commonly used scale [30]. It indicates that the NMS assessed by other scales are not taken into consideration in such trials regardless of their probable influence on the results. Even though TWSTRS analyse motor symptoms extensively, it captures only 30% of symptoms overall.

BFMDRS has the lowest mean overlap (0.09), simultaneously having very weak or weak overlap with scales analysing mostly motor symptoms (e.g. TWSTRS) or NMS (e.g. DNMSQuest). Therefore, this scale should not be used alone but with e.g. Tsui scale or CDSS which evaluate most of motor symptoms while not considering disability assessed in BFMDRS. Instruments with very weak overlap, especially with an overlap which equals zero (e.g. Tsui scale and BFMDRS, Tsui scale and CDQ-24, CDQ-24 and CDSS) should not be used interchangeably as they examine entirely different symptoms.

Taking into consideration our original analysis of symptom categories, three types of scales might be identified – analysing motor symptoms, NMS or both. The first type consists of TWSTRS, Tsui scale and CDSS; the second – CDQ-24 and DNMSQuest; the third – CDIP-58 and BFMDRS. TWSTRS examines the highest number of motor symptoms and disability symptoms; CDIP-58 – of sensory symptoms, sleep disturbances and gait disturbances; CDQ-24 – of emotions and social interactions (however the differences between the number of symptoms analysed by CDQ-24 and CDIP-58 in those categories are small). CDIP-58 is the only scale that examines at least one symptom in each category. The suggested categorization is also supported by specific content overlap analysis. Motor symptoms scales have none or very weak overlap with nonmotor scales, while the overlap score is greater inside the type.

Based on our analysis, CDIP-58 examines the largest number of symptoms and has the highest mean overlap with other scales. Thus, it might be a valuable clinical tool. Furthermore, there is a moderate correlation between the Jaccard index and scale length, and CDIP-58 is the scale consisting of the greatest number of items in our study. However, it analyses a low number of specific motor symptoms. TWSTRS on the other hand is well validated and most commonly used to assess motor symptoms [30]. That is why in our opinion CDIP-58 should be combined with TWSTRS for complex assessment of CD.

### Limitations

As in the previous studies using the content analysis, the main limitation is the number and choice of scales [17, 18]. As before, we implemented a thorough literature search to minimise the risk of bias resulting from wrong scale choice. Another limitation of content overlap analysis is the lack of an objective method to compare the items across the instruments [17, 18]. We followed Fried's (2017) methodology and if there were any doubts about the symptoms we considered symptoms rather too similar than too different [17].

### Conclusions

It was the first study to conduct content overlap analysis of the CD symptoms scales. We showed a very weak overlap among scales and categorised the scales on the basis of assessed symptoms. None of the scales alone examines the CD symptoms exhaustively enough, which is why we recommend combining them.

## Data Availability

The datasets generated during and/or analysed during the current study are available from the corresponding author on reasonable request.
